# 82 Implementing the concept of 24-hour movement behaviours as a determinant of health into the Slovenian environment (GIB24): a national project presentation

**DOI:** 10.1093/eurpub/ckae114.136

**Published:** 2024-09-26

**Authors:** Kaja Kastelic, Tjaša Knific, Nejc Šarabon

**Affiliations:** University Of Primorska, Andrej Marušič Institute, Koper, Slovenia; InnoRenew CoE, Izola, Slovenia; National Institute of Public Health, Ljubljana, Slovenia; InnoRenew CoE, Izola, Slovenia; University of Primorska, Faculty of Health Sciences, Izola, Slovenia

## Abstract

**Purpose:**

Physical activity, sedentary behaviour, and sleep (i.e. 24-hour movement behaviours) are important determinants of health and well-being. Given their perfect co-dependency, there has been a recent recognition that it is sound to address them in a combination. Therefore, the purpose of our ongoing (2023–2025) national project GIB24 is to implement the concept of 24-hour movement behaviours as a determinant of health into the Slovenian environment.

**Project description:**

First, the project will develop Slovenian 24-hour movement guidelines for adults that will integrate recommendations on physical activity, sedentary behaviour, and sleep. The development of the guidelines will follow GRADE-ADOLOPMENT approach, and will include several stakeholders, like kinesiologists, health-care providers, and policy makers. Second, in a collaboration with National Institute of Public Health (NIJZ), we will adapt the national health survey that will allow monitoring of physical activity, sedentary behaviour, and sleep across the whole 24-hour day. The updated items will be implemented in a national survey that will be conducted in 2024. The data collected will allow us to evaluate the adherence to 24-hour movement guidelines among a nationally representative sample of Slovenian adults (n ≈ 9000). Third, a national representative sample (n = 17500) will be invited to participate in a prospective cohort study using thigh-worn activPAL accelerometers. The prospective study aims to estimate prevalence and identify corelates/determinants of device-measured 24-hour movement behaviours among Slovenian adults, and to explore cross-sectional and prospective associations with health and well-being. Fourth, the project will implement the assessment of 24-hour movement behaviours as a vital sign into preventive programmes at the primary health care level.

**Conclusions:**

The project GIB24 will lay foundations for integrated monitoring and promotion of a healthy combination of physical activity, sedentary behaviour, and sleep in Slovenian environment. The evidence-based findings of the project are anticipated to inform future public health policy in Slovenia. The project results will be disseminated in research articles and conferences, popular articles and social media, posters, and flyers. Project dissemination activities are anticipated to raise awareness about the importance of a healthy 24-hour movement behaviours among general, professional, and scientific public.

